# Modern Root Canal Technic Contrasted with the Teachings of One of the Leading Dental Colleges in 1906

**Published:** 1917-07

**Authors:** W. H. Ellis

**Affiliations:** Midnight, Miss.


### MODERN ROOT CANAL TECHNIC CONTRASTED WITH THE TEACHINGS OF ONE OF THE LEADING DENTAL COLLEGES IN 1906

BY W. H. ELLIS, D.D.S., MIDNIGHT, MISS.

The purpose of this article, written wholly at my own volition without suggestion from any one, is to describe the treatment and filling of root canals, in my own individual practice, during the past eight years, compared with the method followed for the first three years after my graduation, the methods of 1906, still being taught very generally.

Immediately after my graduation in 1906, from Vanderbilt, I began practice in the small towns in my state, going from place to place, remaining in each place only so long as I had work to do. In this manner much experience was gained in the smaller towns of the Mississippi Delta, at that time not overburdened with dentists, when even the white people in the towns and for miles around hardly knew what the word ''dentistry'' signified.

At the college we had been taught, and consequently it was our practice, that when a nerve was accidentally exposed, and could be extracted by using a broach, it was good practice to fill the root at once; the theory being that, as it was a fresh wound at the apex, it would heal by ''first intention.'' However, under all other conditions, whether the nerves were destroyed in operation or found dead upon opening up, the root must then undergo a course of treatment before it should be filled, and if there happened to be an abscess, with a fistula, said fistula must be cured and healed perfectly before the root could be filled.

Accordingly, all devitalized teeth were treated in the stereotyped manner, which consisted in first cleansing the root canals, as well as possible, and secondly, in the application of some alleged remedial agent upon cotton in the canal, and filling the cavity with cotton, made more or less waterproof with sandarac varnish.

This canal treatment, or ''remedy'' was Black's 1-2-3. The application was repeated from day to day, or several times a week, according to how many appointments I cared to make with a patient (the amount of work I had to do being taken into consideration), until finally after from six to eight treatments, I filled the canal with chloropercha, if the forceps were not resorted to before that time. And thus, for three years, was my practice conducted. Sometimes, after days of treatment, teeth that I thought ''cured'' would remain in good condition for a few hours only, and then assume a state of sudden and violent activity, bringing great suffering to the patient and trouble to the operator.

If the profession will try to form an idea of the conditions with which I had to contend—the people utterly ignorant of oral hygiene, toothbrushes unobtainable at the few stores, teeth entirely neglected until pain forced the owner of an offending tooth to visit an M.D. and pay him a dollar to ''pull'' it, if it could be done by using one out of an assortment of three or four rusty forceps, the first remark of patient to dentist being invariably, ''How much will it cost me, doctor?''—pulps dead, or nearly so, so badly decayed that one or two scoops with an excavator would expose them; if you can form in your mind a picture of such conditions, where canal treatment was the rule instead of the exception, you will appreciate the joy of my heart in anticipating something modern in the line of treatment for such cases.

Probably forty per cent. of the members of the profession will be unable to appreciate my trials, for they are domiciled in the cities and larger towns of 2,000 to 8,000 inhabitants, with good schools for the little tots, where they are taught oral hygiene from their teachers, the use of the toothbrush, and to notice the attractive displays of dental articles in the drug stores; drawing their clientele from such as these. I say again, this forty per cent. can not appreciate the awful conditions in which I found myself, because preventive treatment in early childhood, constant attention to oral hygiene throughout life, with easy access to the dentist at all times, prevent such cases coming into their practice; but the other sixty per cent. of the profession, the conservative men, in the small villages of 500 to 800 inhabitants, drawing their practice mainly from the country, composed of the class and condition of people that I have described—this greater percentage of my brother dentists will say Amen! They know.

Following these three years of nightmare came eight years of modern practice; comfort to patient, short time of both patient and operator consumed, and last, but by no means least, ninety per cent. of devitalized teeth saved.

In the fall of 1909 I returned to Nashville, and was associated with a very prominent dentist of that city, at that time dean of the dental college, University of Tennessee, as assistant. After a few days with him I had occasion to see him open up and treat a tooth, the pulp of which had died under a very large gold inlay, an upper first molar. The patient had suffered several days until, when he presented himself for treatment, the tooth was very sore, elongated and loose, so much so that the operation of grinding out the inlay was very painful. Finally, after about three-quarters of an hour of pain, the pulp chamber was reached, and the pus boiled up. After thorough drainage, without even attempting to enter the canals, the operator mixed a very thin paste of a material marked on the box "Oxpara," a material that I never had seen or heard of until that day, sealed a quantity of the paste in the pulp chamber, without pressure, with cement so mixed as to drip in—and dismissed the patient for two days. This was sometime in the afternoon, and remembering my "ancient treatment," I remarked to him:

"It is fortunate for that poor fellow that you and I are to return tonight to finish up some laboratory work for early morning, for he is sure to be here, or possibly call you at your home."

He laughed and remarked: "That fellow will be asleep before you and I get back to the office."

He then told me something about the material that he had used; how it would act when sealed in the pulp chamber; how formaldehyde gas would permeate the tubuli of the tooth, through dentin and cementum and beyond, through the apex of the root of the tooth, freeing the protoplasm of every pathogenic germ, sealing its enclosed nuclei forever, positively preventing proliferation.

My answer was, "Yes, sir; very fine indeed; so beautiful! But give that gas and pus sufficient time to accumulate in that absolutely sealed tooth, and we will see what we will see!"

My first remark on entering the office the next morning was: "Did that abscess patient call you last night?"

His answer was: "No, I have heard nothing from him."

"Strange," I replied.

"No, not at all strange. Right at this moment that patient could stand to have a gold filling malleted into that cavity."

Just the same, away down in my heart I thought that some dental parlor man had extracted that tooth some hours since, those men being easiest to reach at night.

Next afternoon patient returned, smoking a cigar and in fine spirits. Upon being questioned, he stated that the tooth had not hurt for a single moment, and was then free from soreness and felt perfectly normal.

Rubber dam and clamp applied, cement removed, Oxpara cleared away and canals entered thoroughly with broach and cleansed. No odor except that of formaldehyde. Oxpara mixed to a little thicker paste this time, canals pumped full of the paste with a thread of cotton on a small broach, a small quantity left in pulp chamber, a cement base placed over this, when hard flattened, and cavity retouched for another gold inlay.

I remained with the doctor thirteen months, saw many cases treated, treated a number myself, and I do not remember of one single extraction on account of abscessed conditions.

I have used the solidified formaldehyde quite extensively, with fine success, but prefer Oxpara, because it is easier to handle, yields any desired thickness of paste, increasing or decreasing the quantity and strength of the medicines, and, in very severe cases, I have found the liquid very helpful. When my treatment is finished, Oxpara gives me a root canal filling, in conjunction with the last treatment when the filling is placed, that so far I have found nothing to equal.

Oxpara neither contracts nor expands. When set and solidified, it is not affected by the fluids of the mouth. It will not dissolve out. This I have tested repeatedly. Again, I have had occasion to enter a bicuspid root canal two and one-half years after treating, for the purpose of putting on a Richmond crown. The odor of Oxpara was still distinctly perceptible—and there was *no other* odor. I did not disturb the filling at the apex; the tooth was in fine condition.

If ever it is necessary to remove an Oxpara filling, a few drops of the liquid on the paste in the pulp chamber will enable the operator to pick the filling out with a broach.

While the directions are plain and simple, there are a few points that should be impressed upon the mind of the operator:

1. Always apply the dam, or keep dry by some other means.

2. Do not try to enter root canals at first sitting in any manner. Use your excavators, without pressure, in removing decomposed tissue and decayed structure. Just place your treatment in the cleansed pulp chamber and seal it in with a soft mix of cement, so as to avoid pressure.

3. Apply the first treatment very thin, to allow gas to be given off. Do not clog up by having the mixture too thick.

4. At second sitting, cleanse canals as thoroughly as possible, but do not thrust a broach into them as you would in placing a piece of cohesive gold. Use small broach, with a gentle, twisting motion, in and out, passing it from side to side so as to contact all of the walls. If there is no odor perceptible except that of the medicine, you are safe in filling at this stage. If there is odor, place another treatment, this time inserting shreds of cotton covered with the paste, and turning them around to cover every part of the canal wall. Seldom have I had to use more than two treatments. The last and final filling of the canals is also a treatment, although the mix should be quite heavy and well spatulated as one would in mixing cement.

It may be necessary to enlarge some of the canals for thorough filling. I often do, and so does every other dentist who tries to do his duty. But, whether you do this with reamers or with a solution of $H_2SO_4$, you can work freely, because you know that you have a sterile field for your operation, from pulp chamber to apex, because the tissues therein and surrounding have been permeated with the gases thrown off by your treatment.

In writing this article I realize that a large percentage of the dental profession have long since stopped trying to cleanse and sterilize root canals, and among them some of our most prominent men in the various associations; but, while I do not believe all that I read about arthritis, rheumatism, etc., being caused by infection from these canals, a glance at some of them that have been passed upon as O. K., and pretty gold crowns, inlays, etc., placed upon and in them, would be sufficient to convince any one that it might well be true. The condition under some of those beautiful restorations might well cause any conscientious man to wonder.

All kinds of statements have been and are being made that all formalin preparations are dangerous antiseptics, that they destroy living cells, cause the parts treated to be more liable to future infection, cause absorption, lessen the density of the alveolus, etc., *"too numerous to mention,"* as they say in the sale bills. These things I do not believe. No drug is dangerous to life as a whole, nor to particular tissues, when used with *discretion*, as all such remedies and drugs should be used. On the other hand, one can, by improper use, and especially by too frequent use, get bad results from any drug or remedy, no matter how harmless it may be when correctly applied. One can produce bad results by the use of iodine, and I have seen gum tissue actually eaten away as the effect of too frequent and too prolonged use. Judgment is what we need.

Of course, situated as I am, I have not access to radiography as a guide; and I wish to state here that I believe that at least sixty per cent. of the profession are in my situation—have not the "advantage" of the X-ray. And I also believe that if they could or would equip themselves for that work, not more than ten per cent. ever would become proficient; but by attempting such work, many deformed jaws would result, not to mention literally millions of teeth that would be lost, for most of their readings would be incorrect.*

I am not decrying radiography as a means to a good end in those very unfavorable cases—pernicious alveolar abscess, blind and discharging; tubercular cryptogamic infections of the peridental membrane, and pericemental abscess in which necrosis of the walls has occurred; but I am contending for a middle ground, from the point of view of a conservative dentist, which I believe that sixty per cent.

---

* The reader is referred to an article by Dr. Charles H. Voelker on this subject, printed in the February number of *The Dental Cosmos*.—EDITOR.

of the dentists of today are, where teeth may be treated and conserved in a rational manner, within the skill of all of us.

In our literature just at this time, nearly every statement made by one writer is flatly contradicted by others. Any of the dental journals of the past few months will bear me out in this. One writer does not believe that placing a guttapercha point through the apical end of a root will cause trouble, gives pictures to prove his contention; the next one takes just the opposite side, gives pictures showing points through the end, crown-pins, etc., causing every known kind of trouble. One refers to "those inoperable canals" as dangerous culture-tubes, while another fills them to perfection; and so on, and on, and on. I could write pages of these so-called "findings" and their causes.

Brothers of the conservative profession, what are we to believe and how are we to proceed? So far as I am concerned, with ninety per cent. of devitalized teeth presenting in ordinary abscessed conditions, of the different varieties, which is the case in any man's practice, I shall continue to do as I have been doing during the last eight years: sterilize, cleanse canals, using best method for the given case, enlarge by the best method for the particular case, and fill the canals to the best of my ability, with Oxpara, with a good cement base covering same in the pulp chamber. That is precisely what I shall do.

As for the other ten per cent. of those very unfavorable cases, presenting conditions such as I have mentioned elsewhere in this article, I shall use my forceps; for I sincerely believe that, from a surgical point of view, any tooth being the direct cause of the troubles that we read about, should be removed from the alveolar socket; for any attempt at root amputation, heroic surgical cutting of surrounding tissues, and excision of one-half to two-thirds of the total length of the root, will leave in the mouth a tooth that can be compared only with some of the poor, maimed soldiers with which the world today is full, so far as any sort of service is concerned.

There are some cases of root amputation, when only a very small piece is removed, to gain or retain a tooth for a pier for some artificial appliance when there is no other recourse, and moderate curettment in these cases, that fall under the head of conservative dentistry, of the conservative-surgical type; but the heroic practices that some would have us believe are common, if daily literature tells the truth, carry the matter entirely too far to receive the endorsement of any conservative man, much less any idea of imitation.

General surgeons amputate and remove diseased, offending organs from the body, and why? Ponder this question, you heroic advocates! and come down to the earth long enough to reflect and see if there is not a surgical limit and a happy medium.—*Dental Digest* for May.

